# Cellular Damage of Bacteria Attached to Senescent Phytoplankton Cells as a Result of the Transfer of Photochemically Produced Singlet Oxygen: A Review

**DOI:** 10.3390/microorganisms11061565

**Published:** 2023-06-13

**Authors:** Jean-François Rontani, Patricia Bonin

**Affiliations:** Aix Marseille Univ, Université de Toulon, CNRS, IRD, MIO UM 110, 13288 Marseille, France; patricia.bonin@mio.osupytheas.fr

**Keywords:** attached bacteria, phytodetritus, oxidative damage, singlet oxygen transfer, solar irradiance, mineral matrices, carotenoids, algal material preservation

## Abstract

Several studies set out to explain the presence of high proportions of photooxidation products of cis-vaccenic acid (generally considered to be of bacterial origin) in marine environments. These studies show that these oxidation products result from the transfer of singlet oxygen from senescent phytoplankton cells to the bacteria attached to them in response to irradiation by sunlight. This paper summarizes and reviews the key findings of these studies, i.e., the demonstration of the process at work and the effect of different parameters (intensity of solar irradiance, presence of bacterial carotenoids, and presence of polar matrices such as silica, carbonate, and exopolymeric substances around phytoplankton cells) on this transfer. A large part of this review looks at how this type of alteration of bacteria can affect the preservation of algal material in the marine environment, especially in polar regions where conditions drive increased transfer of singlet oxygen from sympagic algae to bacteria.

## 1. Introduction

Phototrophic organisms carry out photosynthetic reactions that convert chlorophyll into a singlet excited state (^1^Chl) under the action of light. A small fraction of the ^1^Chl formed can undergo intersystem crossing to produce a longer-living triplet state, ^3^Chl [1]. ^3^Chl can directly damage unsaturated membrane components through type I reactions (i.e., involving radicals) [1], but it can also react with ground-state oxygen (^3^O_2_) to generate singlet oxygen (^1^O_2_) and, although to a lesser extent, superoxide ions (O_2_^−•^). In living cells, the toxic effects of ^3^Chl and ^1^O_2_ are limited by endogenous quenchers or scavengers (carotenoids, tocopherols, ascorbic acid, superoxide dismutase enzymes) [2,3], but this is not the case during cell senescence or cell death. When cells senesce, the slowdown of ^1^Chl consumption in the photosynthetic reactions accelerates the conversion of ^1^Chl into ^3^Chl and, thus, into ^1^O_2_, which then saturates the photoprotective system and ultimately causes photodamage [4,5]. During the senescence of phototrophic organisms, type II photosensitized oxidation processes that mainly involve ^1^O_2_ strongly damage unsaturated membrane lipids. The very high reactivity of ^1^O_2_ with unsaturated compounds results from its strong electrophilicity and the lack of spin restriction that normally hinders ^3^O_2_ reacting with unsaturated compounds [6].

**Figure 1:**
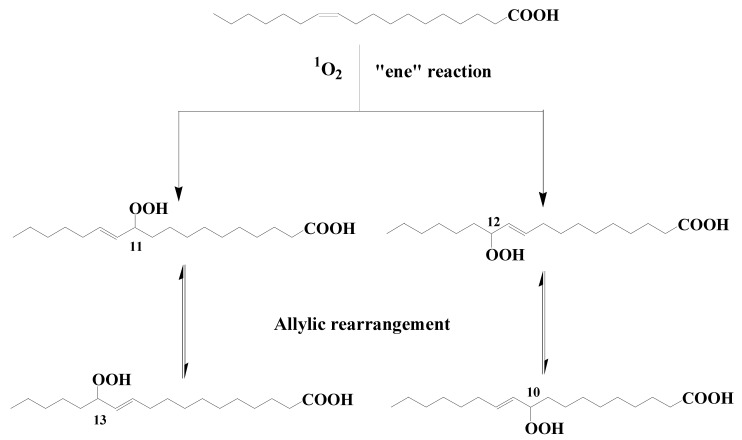
Type II photosensitized oxidation of cis-vaccenic acid and subsequent allylic rearrangement of the hydroperoxyacids formed.

Type II photosensitized oxidation processes act intensively on several unsaturated lipids, including chlorophyll itself but also unsaturated fatty acids, sterols, some *n*-alkenes, some highly branched isoprenoid (HBI) alkenes, carotenoids, and tocopherols (for review, see [5,7,8,9]). Type II photosensitized oxidation of monounsaturated fatty acids (MUFAs) involves a direct reaction of ^1^O_2_ with the carbon–carbon double bond via a concerted ‘ene’ addition [10], leading to the formation of hydroperoxides at each end of the original double bond [11] (see Figure 1 showing oxidation of cis-vaccenic acid). These hydroperoxides, which possess an allylic trans-double bond can subsequently undergo stereoselective radical allylic rearrangement and afford two other isomers with a trans-double bond [11,12] (Figure 1). Type II photosensitized oxidation of MUFAs, thus, leads to the formation of four isomeric allylic hydroperoxyacids, which are generally converted to their corresponding hydroxyacids by NaBH_4_ reduction for quantification in natural samples [13]. This approach has been used to detect high levels of photoproducts of phytoplanktonic MUFAs in several marine particulate and sediment samples (for a recent review see [9]).

Surprisingly, photoproducts of octadec-11(cis)-enoic acid (cis-vaccenic acid) have also been detected in a number of different zones of the oceans [7,8,9,14,15] and sometimes in similar or even higher proportions (relative to the parent acid) than photoproducts of phytoplanktonic MUFAs. Cis-vaccenic acid has been proposed as a useful biological marker for bacteria in the marine environment, based on its higher relative concentrations in bacteria than in other organisms [16,17,18,19,20]. However, given that heterotrophic bacteria lack photosynthetic system, the presence of high proportions of these photoproducts was a very surprising finding.

Here we summarize and review the findings of the research that has been carried out to determine: (i) the phenomenon at the origin of this oxidation of cis-vaccenic acid observation, (ii) the parameters that promote the oxidation of bacteria, and (iii) the impact of the oxidation state of bacteria on the preservation of phytoplanktonic organic matter in the oceans.

## 2. Potential Sources of Photoproducts of cis-Vaccenic Acid in Oceans

### 2.1. Photooxidation of Aerobic Anoxygenic Phototrophic Bacteria (AAPB)

Aerobic anoxygenic phototrophic bacteria (AAPB) are an important group of microorganisms inhabiting the euphotic zones of oceans and freshwater or saline lakes [21]. They do not form a monophyletic clade but are widely distributed within Alphaproteobacteria, Betaproteobacteria, and Gammaproteobacteria classes [22,23]. These bacteria perform a heterotrophic metabolism because they require organic carbon for growth, but they can also use photosynthesis as a supplemental energy source [24]. Due to their ability to obtain extra energy from light, AAPB can have a higher impact on the degradation of organic matter than strict heterotrophs [25]. It has been previously demonstrated that AAPB are widely distributed in the open ocean [26,27,28]. The induction of type II photosensitized oxidation processes in these organisms, which contain bacteriochlorophyll (which is a highly efficient photosensitizer [29]) and high proportions of cis-vaccenic acid in their membranes [30,31] could, therefore, be at the origin of the presence of cis-vaccenic acid photoproducts in the samples investigated.

To test this hypothesis, senescent cells of *Erythrobacter* sp. strain NAP1 and *Roseobacter* sp. strain COL2P were exposed to photosynthetically available radiation (PAR) [32]. The profile of oxidation products of cis-vaccenic acid obtained after exposure to PAR did not correspond to the profile observed in situ. In fact, as we have seen previously, the attack of cis-vaccenic acid by ^1^O_2_-mediated processes only produces trans allylic hydroperoxyacids (Figure 1), whereas the irradiation of senescent AAPB results in the formation of high proportions of cis isomers, which are characteristic of radical oxidation processes [33]. Previous research shows that the ^1^O_2_ produced in senescent phytoplankton cells (for a review see [9]), but also in purple sulfur bacteria (*Thiohalocapsa halophila* and *Halochromatium salexigens*) [34] by type II chlorophyll or bacteriochlorophyll-photosensitized processes intensively attacks the phytyl side-chain of these pigments, affording a specific photoproduct (3-methylidene-7,11,15-trimethylhexadecan-1,2-diol) [8]. The fact that this compound is not found after irradiation of AAPB confirms that ^1^O_2_ is only very weakly produced during the senescence of these organisms. The oxidation of cis-vaccenic acid in AAPB, thus, appears to involve radical degradation processes and is clearly not at the origin of the presence of high proportions of cis-vaccenic acid photooxidation products resulting from ^1^O_2_-mediated processes in the oceans.

### 2.2. Transfer of Photochemically Produced ^1^O_2_ from Senescent Phytoplanktonic Cells to Their Attached Heterotrophic Bacteria

**Figure 2:**
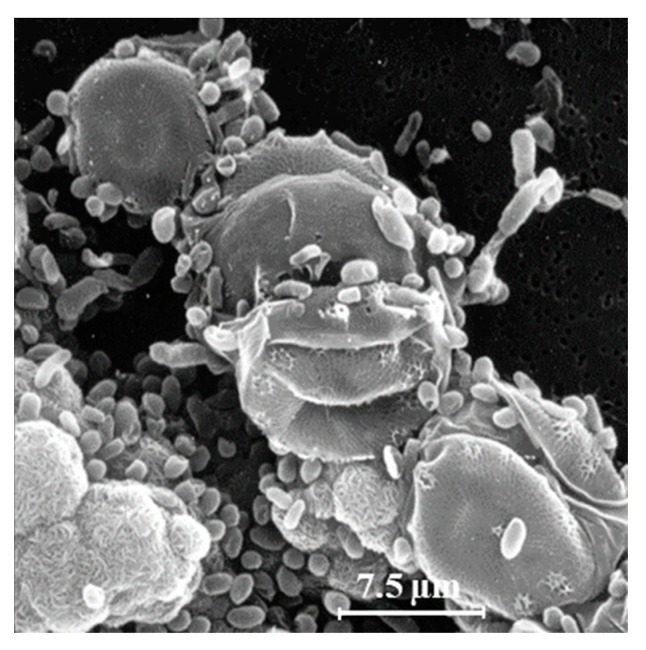
Scanning electron microscopy image of a strain of the diatom *Skeletonema costatum* colonized with heterotrophic bacteria (adapted from [32]).

Another explanation for the unexpected presence of cis-vaccenic acid photoproducts in marine systems could be ^1^O_2_ transfer in attached heterotrophic bacteria during the senescence of phytoplankton. Bacteria are known to colonize phytoplankton-derived particles [35,36]. Figure 2 shows an example of the close association between bacteria and phytoplankton. Senescent phytoplanktonic cells provide hydrophobic micro-environments in which the lifetime and the potential diffusive distance of ^1^O_2_ could be long enough to induce type II photosensitized oxidation processes in attached bacteria. Indeed, the intracellular sphere of activity of ^1^O_2_ has recently been re-evaluated [37] and the radius of this sphere of activity from the point of production appeared to be larger than previously thought. It is estimated at between 155 and 340 nm [37,38,39], which is a large-enough distance to allow ^1^O_2_ to cross the cell membranes of phytoplanktonic cells (thickness ranging from 70 to 80 nm) [37,40,41] and, thus, reach attached bacteria.

In an attempt to validate this hypothesis, Rontani et al. [32] performed parallel experiments using PAR lamps to irradiate: (i) dead axenic cells of the diatom *Skeletonema costatum* strain CS-181, (ii) dead axenic cells of the same diatom contaminated with a heterotrophic bacterial community, and (iii) the heterotrophic bacterial community (that had been used as the contaminant community) alone [37,38]. The results obtained showed that the photodegradation of cis-vaccenic acid from heterotrophic bacteria was more than two orders of magnitude faster in heterotrophic bacteria attached to phytoplanktonic cells than in the bacterial community alone. Interestingly, the profile of the cis-vaccenic oxidation products obtained matched perfectly to the profile detected in situ. This means that ^1^O_2_ produced by type II photosensitized oxidation processes in senescing phytoplanktonic cells can migrate across the membranes to the attached heterotrophic bacteria and go on to induce oxidative damage in them. These results were later confirmed by Petit et al. [42], who show that the photodegradation state of cis-vaccenic acid from bacteria attached to phytodetritus is strongly correlated with the photodegradation state of their chlorophyll. Photodegradation of heterotrophic bacteria attached to senescent phytoplanktonic cells, thus, emerges as the likely source of the oxidation products of cis-vaccenic acid detected in situ. This assumption is well-supported by the fact that attached bacteria are more likely to become part of the sinking material (which also shows strong photooxidation of cis-vaccenic acid) [7,13] than free-living AAPB. 

## 3. Effect of Polar Matrices Surrounding Phytoplankton Cells on the Transfer of ^1^O_2_ from Irradiated Phytodetritus to Their Attached Bacteria

### 3.1. Silica and Carbonaceous Charged Mineral Surfaces

Previous research shows that ^1^O_2_ has a longer lifetime and greater potential diffusion distance in hydrophobic environments than in hydrophilic environments [43]. Hurst and Schuster [44] subsequently show that: (i) the shortest lifetimes are observed in solvents possessing O-H groups, particularly water, and (ii) the presence of heavy atoms reduces the lifetime of ^1^O_2_. ^1^O_2_ transfer is strong between two lipophilic membranes (such as those of phytoplankton and associated bacteria) [42], but this excited form of oxygen could rapidly be deactivated if the two membranes are separated by frustules or coccoliths. Indeed, diatoms build a rigid cell wall made of amorphous silica (frustules) containing O-H groups and aluminum [45,46,47], while coccolithophorids, which belong to the algal class Prymnesiophyceae, are able to produce scales made of CaCO_3_ called coccoliths [20,48].

Petit et al. [49] previously compared the ^1^O_2_-induced damages to attached bacteria during the irradiation of dead cells of non-axenic *Emiliania huxleyi* strain RCC1215 (a prymnesiophyte with coccoliths), *Skeletonema costatum* strain RCC70 (a diatom with a silica matrix), *Navicula jeffreyi* strain CS513 (another diatom with a silica matrix), and *Dunaliella tertiolecta* strain RCC6 (a chlorophyte without a matrix). The results show that the presence of diatom frustules inhibits the transfer of ^1^O_2_ to the attached bacteria, whereas the presence of coccoliths has no effect. The authors attribute the lack of effect of coccoliths to the fact that they are released during cell senescence [50,51], allowing efficient transfer of ^1^O_2_ to the attached bacteria.

**Figure 3:**
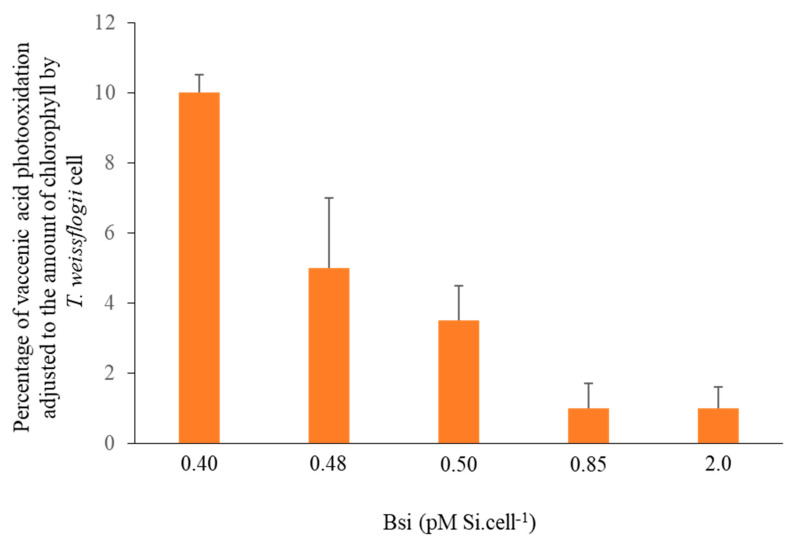
Graph plotting percentage of cis-vaccenic acid photooxidation products adjusted to concentration of chlorophyll a per *Thalassiosira weissflogii* cell according to concentration of biogenic silica (pmol cell^−1^) (adapted from [49]).

Petit et al. [49] also show that, in the case of diatoms, the percentage of cis-vaccenic acid photooxidation is inversely correlated with biogenic silica concentration (Figure 3). Note that the limitation of ^1^O_2_ transfer in the marine diatom frustules may be attributed not only to the presence of O-H groups or aluminum atoms [44], but also to (potentially antioxidant) mycosporine-like amino acids [52,53], which are often present in diatom frustules [54,55].

### 3.2. Exopolymeric Substances (EPS)

Algae produce EPS to: (i) promote the formation of microalgal aggregates, (ii) facilitate cell adhesion to a substrate that serves to form a biofilm matrix, (iii) release metabolic-excess waste products, and/or (iv) help protect cells against dewatering and toxic substances [56,57,58]. In addition, EPS can act as energy and carbon sinks in response to stress [56]. Microalgal EPS are mainly composed of exopolysaccharides, proteins (enzymes and structural proteins), nucleic acids (DNA), and lipids [56,57].

It was recently suggested that EPS may also—due to their hydrophilic nature—reduce ^1^O_2_ diffusion distance and, thus, inhibit ^1^O_2_ transfer to bacteria [59]. To illustrate the variation in the photo-oxidation of attached bacteria relative to the photooxidation of algae, Amiraux et al. [59] plotted the percent photo-oxidation of cis-vaccenic acid against the percent photo-oxidation of 24-methylenecholesterol (algal sterol) [60] in several sediment trap samples collected at 5 m and 30 m depth in the Canadian Arctic. They observed that ^1^O_2_ transfer from phytodetritus to attached bacteria was less efficient in the deeper sinking particles, which they attributed to the higher aggregated state of the ice algae in these samples and, thus, a higher concentration of EPS inhibiting ^1^O_2_ transfer from senescent algae to their attached bacteria. Note, however, that the lower efficiency of ^1^O_2_ transfer observed in the deeper trap may also be due to the natural decrease in solar irradiance with depth (see next section). 

## 4. Effect of Solar Irradiance Intensity on the Transfer of ^1^O_2_ from Irradiated Phytodetritus to Their Attached Bacteria

It has been previously demonstrated that low solar irradiance favors slower production and diffusion of ^1^O_2_ across the cell membranes of phytoplankton and, thus, greater photo-oxidative damage to the unsaturated lipids in senescent phytoplankton rather than chlorophyll photodegradation (sensitizer photobleaching) [8,61]. A very recent study investigated the effect of solar irradiance intensity on the transfer of ^1^O_2_ from phytodetritus to their associated bacteria [41]. 

**Figure 4:**
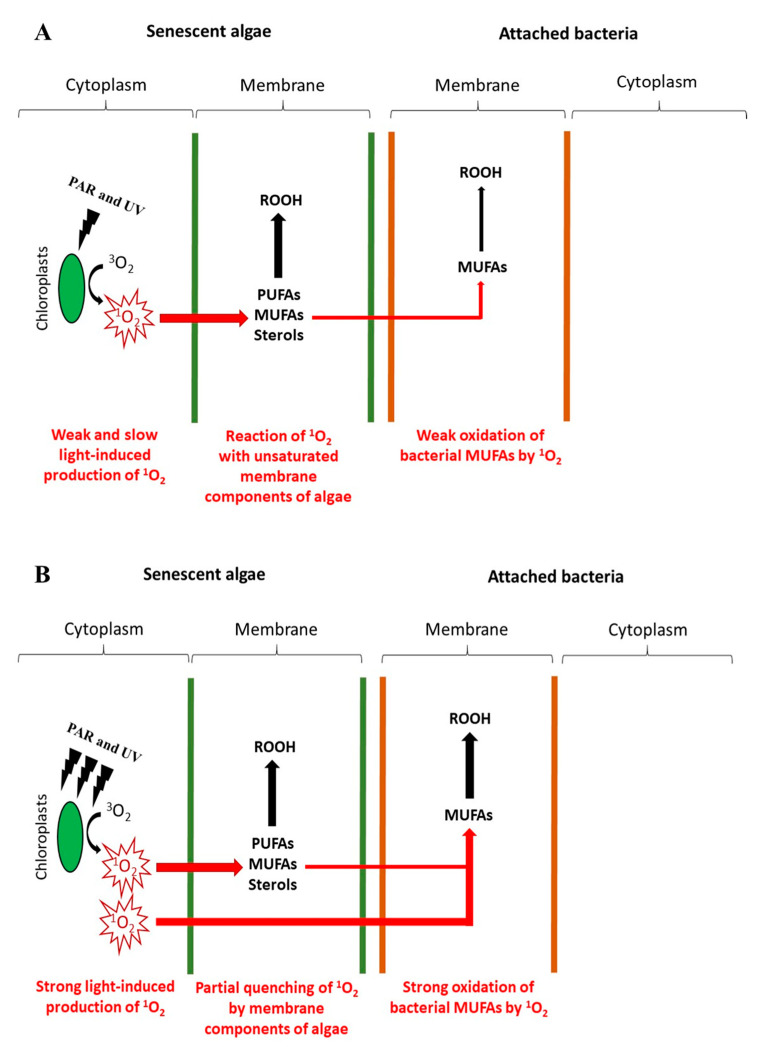
Conceptual schemes showing the transfer of ^1^O_2_ from senescent phytoplankton cells to the membranes of their attached bacteria under (**A**) low and (**B**) high solar irradiances (adapted from [41]).

Irradiation of senescent cells of the diatom *Thalassiosira* sp. in association with the bacterium *Pseudomonas stutzeri* under contrasted artificial light irradiances shows that oxidative damage induced by ^1^O_2_ in bacterial membranes increases with irradiance [41]. Indeed, at low irradiances, the ^1^O_2_ that is slowly produced in phytoplanktonic chloroplasts reacts intensively with unsaturated lipids in the algal membrane (photodynamic effect) and is, thus, quenched before it can reach bacterial membranes (Figure 4A). Conversely, high irradiances induce a rapid and intense production of ^1^O_2_ that is only partially consumed in phytoplanktonic membranes and easily reaches the attached bacteria, where it efficiently oxidizes their unsaturated membrane components (Figure 4B). Further analysis of numerous sinking particle samples collected from different regions of the Canadian Arctic confirmed these in vitro results [41]. The photo-oxidation state of attached bacteria increased on a gradient from ice-covered areas to open water (i.e., from low-irradiance to high-irradiance areas). Interestingly, photo-oxidation of bacteria appeared to be particularly intense in bacteria attached to sympagic (i.e., associated with sea ice) algae [41]. This very strong photo-oxidation state has been attributed to the fact that the sympagic algae–bacteria association in sea ice is maintained at relatively high irradiances (up to 106 µmol photons m^−2^ s^−1^ after snowmelt) [62] for relatively long periods of time.

## 5. Effect of Bacterial Carotenoid Content on the ^1^O_2_ Transfer from Phytodetritus to Attached Bacteria

Carotenoids extent the wavelength range of light that is able to drive photosynthesis by transferring their absorbed energy (in the blue–green region of the solar spectrum) to chlorophylls or bacteriochlorophylls [63,64,65]. Carotenoids also play a major photoprotective role in photosynthetic organisms by quenching or scavenging excess ^3^Chl and ^1^O_2_ [5,62]. These compounds are, therefore, widely distributed in phototrophic bacteria (including cyanobacteria, purple bacteria, green sulfur bacteria, and AAPB) [66,67]. Note, however, that some non-phototrophic bacteria have acquired carotenogenic genes, enabling them to use these compounds as protection during the events of intense stress [68,69,70]. 

Petit et al. [71] monitored the dynamics of the bacterial community attached to irradiated cells of the Prymnesiophyte *E. huxleyi* and showed that in late stationary phase more than 90% of attached bacteria were dead. Interestingly, the remaining 10% of live bacteria appeared to be dominated by pigmented species (*Maribacter*, *Roseobacter*, *Roseovarius*), suggesting that carotenoids play a major role in bacterial resistance to ^1^O_2_ stress. Indeed, it has previously been hypothesized that bacteria containing high amounts of carotenoids might be able to tolerate exposure to ^1^O_2_ [72,73], but this assumption has never been confirmed in the case of bacteria attached to irradiated phytodetritus.

Recent research [41] investigated the effect of ^1^O_2_ produced during the senescence of a widespread diatom (*Thalassiosira* sp.) on two attached Gram-negative bacteria widely found in marine environments, i.e.: *Pseudomonas stutzeri* [74] (a heterotrophic bacterium that does not contain carotenoids) and *Dinoroseobacter shibae* [75], which is an AAPB that contains the carotenoid spheroidenone. The originality of this work was that it investigated the effect of ^1^O_2_ produced during the senescence of this diatom on the physiology of pigmented and non-pigmented bacteria associated with it at both membrane–lipid level and DNA level. Indeed, in cells, ^1^O_2_ reacts not only with unsaturated membrane lipids and proteins [76,77] but also with nucleic acids [77,78], where it reacts mainly with the guanine nucleobase to form 8-oxo-7,8-dihydro-2′-deoxyguanosine (8-oxodG) [79], which induces various mutations [80]. 

Bacterial cells use several strategies to protect themselves against the toxicity of ^1^O_2_: (i) detoxification enzymes, such as superoxide dismutase or catalase [81,82]; (ii) an efficient “Go system” with three detoxification proteins, i.e., MutM, MutT, and MutY [83,84], which is involved in DNA repair; and (iii) quenching ^1^O_2_ with carotenoid pigments [85].

**Figure 5:**
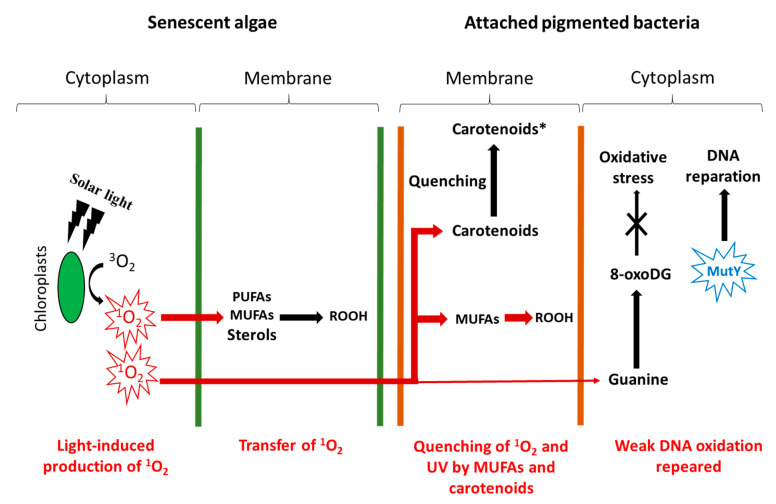
Conceptual scheme showing the transfer of ^1^O_2_ from senescent phytoplankton cells to their attached bacteria in the presence of bacterial carotenoids (adapted from [41]) (carotenoids* corresponds to the excited state of carotenoids).

Burot [41] observed that the presence of spheroidenone in *D. shibae* limits but does not completely prevent ^1^O_2_-induced oxidative alterations of unsaturated membrane lipids. However, by monitoring the activation and regulation of the DNA repair system and the *rpoH* gene responsible for the oxidative stress response [86,87], Burot [41] shows that due to the quenching and scavenging activity of spheroidenone and MUFAs in the bacterial membranes, only a small fraction of ^1^O_2_ actually reaches the cytoplasm, where the efficient detoxifying activity of *mutY* limits its impact on the DNA of this strain and, thus, prevents oxidative stress (Figure 5). Conversely, in *P. stutzeri* cells, the scavenging activity of membrane MUFAs and the DNA repair system are not sufficient to prevent DNA damage and oxidative stress in the cytoplasm. 

Note, however, that 8-oxoDG is not specific to ^1^O_2_-induced alteration of DNA, but is also produced during the oxidation of DNA by other reactive oxygen species (ROS) (e.g., peroxyl or hydroxyl radicals arising from hydroperoxide homolysis or H_2_O_2_) [88,89,90]. Consequently, the alteration of DNA observed in bacterial cytoplasm may also result from the action of these ROS (notably by H_2_O_2,_ which is known to readily cross cell membranes [91,92]. 

Marine phytoplankton-associated bacterial communities are often dominated by *Roseobacter*, a genus belonging to the Alphaproteobacteria class [93,94,95,96,97]. The ability of these bacteria to colonize algal blooms has been attributed to (i) their high colonization capability [98], and (ii) their ability to produce quorum-sensing molecules [99] or antimicrobial compounds [100]. The results of Burot [41] allow us to propose another explanation for the dominance of *Roseobacter* in algal blooms, which is that their carotenoid content enables them to resist the flux of ^1^O_2_ from senescent phytoplankton cells.

## 6. Effect of the Production of ^1^O_2_ by Irradiated Phytodetritus on Motile Bacteria

Motile bacteria that are challenged by physical or chemical stimuli can move towards more favorable conditions to exploit new resources or opportunities [101,102]. This bacterial chemotaxis provides an important competitive advantage in terms of accessibility to inert particles or living organisms (such as microalgae). They are potentially several phytoplankton–bacteria interactions that can all co-exist [103,104,105], and the biotic preservation of phytodetritus is ultimately determined by the resulting balance between attractant and repulsive effects [106,107].

To determine whether or not the production of ^1^O_2_ by phytodetritus can repel motile bacteria, a chemotaxis experiment was performed with the bacterium *Shewanella oneidensis* (chosen for its well-known chemotactic capacity) [107,108]. The results obtained showed a strong attractant effect of phytodetritus (dead *E. huxleyi* cells) regardless of whether they were irradiated [71]. The observed lack of repulsive effect was attributed to: (i) the lack of sensors that would allow *S. oneidensis* to detect ^1^O_2_, (ii) an attractive effect of phytodetritus surpassing the putative repulsive effect of ^1^O_2_, or (iii) the fact that ^1^O_2_ has very short lifetime in water, which substantially limits its diffusion distance (0.1–0.2 μm) [109]. Petit et al. [71] also observed a very high proportion (90%) of dead attached bacteria on the phytodetritus and hypothesized that bacteria that are unable to detect ^1^O_2_ production but strongly attracted by senescent phytoplanktonic cells could accumulate on them and then be killed by the ^1^O_2_ transfer. Further experiments are needed to confirm this interesting hypothesis and to determine whether the inability of *S. oneidensis* to detect ^1^O_2_ production can effectively be extended to other bacterial assemblages attached to phytodetritus.

## 7. Induction of Autoxidative Processes in Bacteria: A Consequence of Photooxidation Processes

Spin restriction [110] means that the unpaired electrons of ground-state triplet molecular oxygen (^3^O_2_) can only interact with unpaired electrons of transition metals or organic radicals. Autoxidation, thus, involves free-radical-mediated oxidation chain reactions, which can be divided into three steps: initiation, propagation, and termination [111]. Initiation, which is the crucial first step in these processes, requires initiators or catalysts that are able to generate radicals (by removing an electron or breaking a weak covalent bond) and, thus, start the chain reactions.

**Figure 6:**
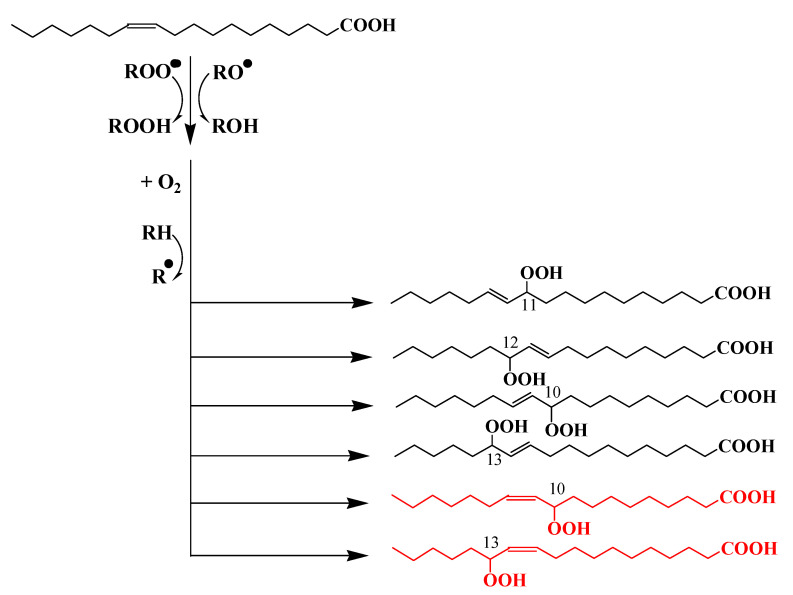
Autoxidation of cis-vaccenic acid initiated by peroxyl and alkoxyl radicals arising from the homolytic cleavage of photochemically produced hydroperoxides (in red: the cis-hydroperoxyacids, which are specific tracers of autoxidation processes) (adapted from [11,33]).

Hydroperoxides resulting from ^1^O_2_-induced oxidation of unsaturated membrane components of bacteria (e.g., MUFAs) (Figure 1) are relatively unstable (O–O bond dissociation energy = 34 kcal/mol) and can, thus, be readily cleaved by heat, light, some redox-active metal ions undergoing one-electron transfer (e.g., Fe^2+^, Co^2+^, Fe^3+^, Cu^2+^, Mn^2+^, Zn^2+^, Mg^2+^, V^2+^), and certain enzymes (e.g., lipoxygenases) to hydroxyl, peroxyl, and alkoxyl radicals (for review, see [8,111]. In oxic environments, these radicals can then induce autoxidation (i.e., radical chain oxidation) of MUFAs. These processes involving allylic hydrogen abstraction and subsequent oxidation of the allylic radicals formed afford a mixture of six isomeric cis and trans allylic hydroperoxides [9,33]. In the case of cis-vaccenic acid, autoxidation produces 11-hydroperoxyoctadec-12(trans)-enoic acid, 12-hydroperoxyoctadec-10(trans)-enoic acid, 10-hydroperoxyoctadec-11(trans)-enoic acid, 10-hydroperoxyoctadec-11(cis)-enoic acid, 13-hydroperoxyoctadec-11(trans)-enoic acid, and 13-hydroperoxyoctadec-11(cis)-enoic acid (Figure 6). cis-Allylic hydroperoxy acids are specific to autoxidation processes [33] and, thus, it makes it easy to detect these processes in environmental samples [9,14]. 

Examination of several particulate matter and sediment samples revealed the presence of varying proportions of 13-hydroperoxyoctadec-11(cis)-enoic acid and 10-hydroperoxyoctadec-11(cis)-enoic acid among the oxidation products of cis-vaccenic acid [7,14,15,112], which confirms the involvement of autoxidation processes in bacteria attached to particles. A study comparing the oxidation state of cis-vaccenic acid in particulate matter samples and in the underlying surficial sediments collected in Baffin Bay in the Arctic [112] shows that bacteria attached to sinking particles are mainly photo-oxidized whereas bacteria present in the underlying sediments are strongly autoxidized. These interesting results clearly establish that bacteria associated with sinking algal material are strongly affected by the ^1^O_2_ photochemically produced in senescent algae during their transfer through the euphotic layer of the water column of the oceans. These bacteria are then subjected to intense autoxidation during their stay in the oxic layer of sediments. The radicals at the origin of this intense autoxidation are probably derived from the degradation in oxic sediments of the labile hydroperoxides photochemically produced in the water column. However, it should be noted that the incorporation of oxidized free fatty acids (FFA) excreted by sympagic algae in bacterial membranes may also play a role in the induction of autoxidation processes in attached bacteria [113].

## 8. Impact of the Oxidation of Bacteria Attached to Microalgal Material on Algal Preservation: A Focus on the Arctic

Diatoms, dinoflagellates, and coccolithophores are the main primary producers in marine ecosystems that are capable of using light energy and inorganic nutrients to produce organic matter (OM) [114]. It is generally considered that approximately 50% of the marine primary production (PP) is mineralized by bacteria [115]. The remaining 50% of PP either enters the marine food web or is buried in sediments through a process called the ‘biological pump’ [116]. Only a small fraction of the OM produced within the upper water column reaches the sediments where it can contribute to CO_2_ storage; one study put this fraction at just 1% of the OM originally produced [117]. However, the amount and composition of OM preserved in marine sediments varies greatly between different regions and depositional environments [118,119]. Indeed, organic carbon preservation is mediated by several parameters, including: (i) oxygen concentration [120], (ii) sedimentation rate [121], (iii) protection through interactions with a mineral matrix (mainly clays and iron oxides) [119,122], (iv) physiological status of the bacterial communities associated with sinking particles [59], and (v) match or mismatch of zooplanktonic grazing with algae fluxes [123].

**Figure 7:**
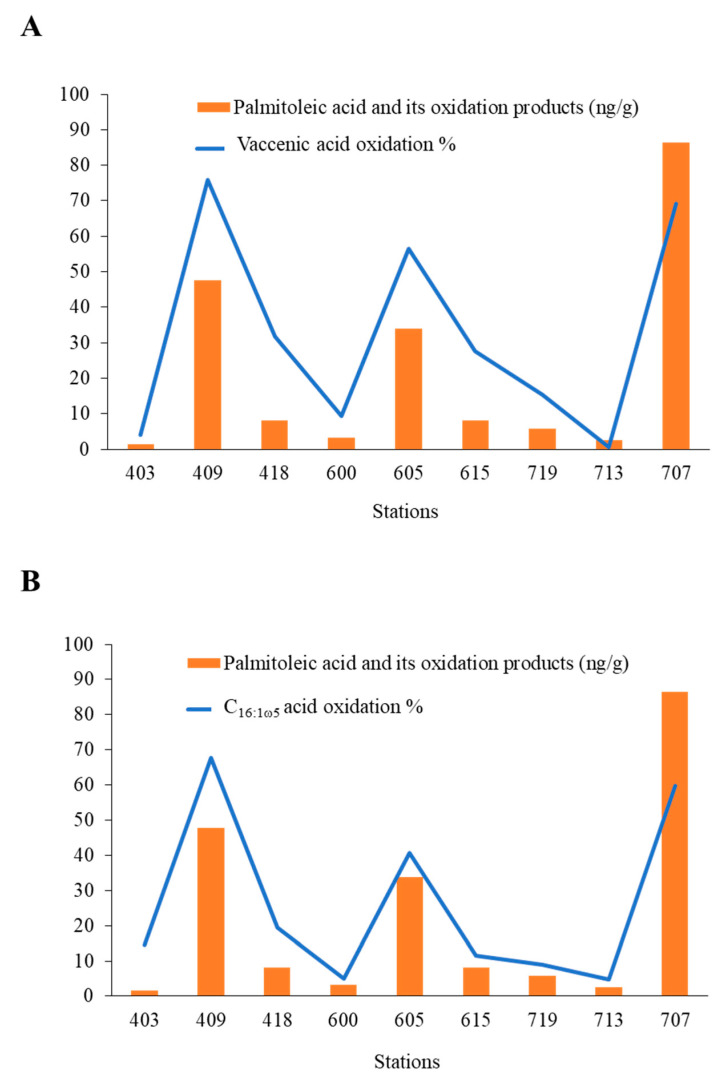
Concentration of palmitoleic acid and its oxidation products (ng g^−1^) (indicative of sympagic algal material abundance) and percent oxidation of (**A**) cis-vaccenic acid and (**B**) C_16:1ω5_ acid (indicative of bacterial damage) measured by Rontani et al. [112] in surficial sediments (0–1 cm) sampled from a set of stations investigated in central and eastern Baffin Bay (Canadian Arctic).

In the Arctic, sympagic algae are assumed to be one of the main sources of organic matter reaching the seafloor [59,124,125], as they strongly aggregate (due to the high concentrations of EPS produced by these organisms in the ice) and, thus, sink faster than pelagic algae [126]. Moreover, the bacteria associated with them are in a weak physiological state and, thus, have only weak mineralization capabilities [59,123]. Indeed, in ice, these bacterial communities are strongly altered by: (i) intense osmotic stress induced by salinity changes in brine channels during the early stages of ice melt [59], (ii) production of bactericidal FFA and hydroperoxides by sympagic algae in response to light stress [112,123], and (iii) intense transfer of ^1^O_2_ from sympagic algae [41] (also see Section 5). We recently examined the lipid content of surficial sediments (0–1 cm) and sinking particles collected in summer from central and eastern Baffin Bay during the 2016 GreenEdge campaign [112]. Yunda-Guarin et al. [125] previously suggested that most of the organic carbon present in these sediments arises from sympagic algae. Sympagic algal preservation can be monitored in sediments by using the concentration of intact and oxidized C_16:1ω7_ (palmitoleic) acid. Given the dominance of diatom biomass (compared to bacteria) in the Arctic, palmitoleic acid is generally considered to be a robust marker of primary producers in this region [127]. In parallel to measuring the concentrations of intact and oxidized palmitoleic acid, we paid particular attention to the oxidation state of cis-vaccenic and C_16:1ω5_ acids (bacterial fatty acids) [128,129]. Some of the sediments investigated showed strong autoxidation of sympagic algae and their attached bacteria (Figure 7) [112]. 

Autoxidation of bacteria likely results from a transfer of ^1^O_2_ from senescent sympagic algae to their attached bacteria in ice and in the euphotic layer of the water column, followed by subsequent induction of radical chain oxidation by homolysis of the hydroperoxides formed in the underlying sediments [8,130]. This assumption is well-supported by the strong photooxidation state of bacteria observed in sinking particles that were also collected during the 2016 GreenEdge campaign [112]. Note that palmitoleic acid concentration (i.e., a marker of the preservation of sympagic algal material) [127] appeared to be highest at the stations containing strongly oxidized (and, thus, inactive) bacteria (Figure 7). These observations clearly establish the link between the degree of oxidative alteration of bacteria and the efficiency of biodegradation processes. The oxidative stress induced in attached bacteria by the transfer of ^1^O_2_ from senescent algal cells and the subsequent autoxidation reactions must, therefore, play a key role in the sedimentary preservation of algal material, particularly in the case of sympagic algae due to the enhancement of ^1^O_2_ transfer in ice [41].

Benthic bacteria that are well-adapted to the deep-sea environment are generally thought to be the major contributors to the degradation of algal material in sediments [131]. In the Arctic, these benthic bacteria are dominated by members of the *Roseobacter* clade [132], which is known to contain high levels of cis-vaccenic acid [29,133]. The very strong oxidation of cis-vaccenic acid observed in some of the sediments investigated by Rontani et al. [112] (Figure 7A) suggests that deposited ice algal aggregates escape colonization by active benthic bacteria. This surprising observation is attributed to the bactericidal properties of the hydroperoxides [134,135] and FFA [136], which are found in high proportions in sympagic algal material [112]. 

^1^O_2_ transfer from senescent algal cells in sea ice and the euphotic layer of the water column and the subsequent autoxidation reactions in oxic sediments reduce the mineralization capabilities of bacteria associated with the sympagic algal material and, thus, favor the preservation of this last one [112]. Furthermore, the bactericidal properties of the hydroperoxides resulting from oxidation processes shield sympagic algal material against colonization by active benthic bacteria and, thus, also contribute to better preservation of algal material in Arctic surficial sediments. 

In the Arctic Ocean, carbon fluxes within the biological pump appear to be sensitive to climate perturbations. Indeed, primary production in the Arctic Ocean is supported by sympagic algae during the ice-covered period and then by pelagic phytoplankton in open waters. Due to the effects of global warming (reducing the extent and duration of sea ice), we are currently witnessing a decline in the contribution of sympagic algae to primary production. Unfortunately, as these algae are assumed to be one of the main sources of OM reaching the seafloor [59,61,123,124], the biological pump may act as a positive feedback loop for global warming.

## 9. Future Research Developments

Future studies dealing with the preservation of phytoplanktonic material in sediments should be designed to take into account the photo- and autoxidative alteration of bacteria associated with this material. This would make it possible to better understand, accurately estimate, and, thus, better anticipate how phytoplankton degradation/preservation is likely to respond to climate change. 

Future research should also pay special attention to the study of interactions between biotic and abiotic degradation processes, which have not been sufficiently considered in the literature. It is very important to not consider these processes separately but to consider their interactions, which, as we have shown in this review, can have major biogeochemical consequences.

## 10. Conclusions

The results of the different studies summarized in this review show that when the senescence of phytoplankton occurs under high solar light irradiances, the ^1^O_2_ photochemically produced in chloroplasts can efficiently migrate across phytoplankton membranes to the attached bacteria and it can cause intense oxidative damage. This process, which is enhanced in sympagic algae, appears to be central to the preservation of algal material in the Arctic by limiting the mineralization capabilities of the phytodetritus-associated bacteria in the water column. Moreover, in surficial sediments, hydroperoxides produced by photo-oxidation and autoxidation processes in algae also limit the colonization of phytodetritus by active benthic bacteria.

## Data Availability

Not applicable.
